# The complete chloroplast genome sequence of *Vincetoxicum mongolicum* (Apocynaceae), a perennial medicinal herb

**DOI:** 10.1590/1678-4685-GMB-2022-0303

**Published:** 2023-06-02

**Authors:** Wangsuo Liu, Zhanjun Wang, Ying Tian, Bo Ji

**Affiliations:** 1 Institute of Forestry and Grassland Ecology, Ningxia Academy of Agriculture and Forestry Science, Yinchuan, Ningxia, China.; 2 Ningxia Technical College of Wine and Desertification Prevention, Department of hydraulic engineering, Yinchuan, China.; 3 Ningxia Academy of Agriculture and Forestry Science, Ningxia Key Laboratory of Desertification Control and Soil and Water Conservation, Yinchuan, Ningxia, China.

**Keywords:** Vincetoxicum mongolicum, complete chloroplast genome, Apocynaceae, comparative analysis,, phylogenetic tree

## Abstract

*Vincetoxicum mongolicum* Maxim. (1876), is a perennial medicinal herb, widely distributed in the Loess Plateau of China. Here, we sequenced, assembled, and annotated the complete chloroplast (cp) genome of *V. mongolicum*, and compared the highly variable gene regions and phylogenetic positions between *V. mongolicum* and other related species. Results showed that the complete cp genome of *V. mongolicum* was 160,157 bp in length, containing a large single copy (LSC) region of 91,263 bp, a pair of inverted repeats (IR) region of 23,892 bp, and a small single copy (SSC) region of 21,110 bp. The GC content accounts for 37.8%, and we annotated 131 single genes, which include 86 protein-coding genes, 8 rRNA genes, and 37 tRNA genes. By comparing and analyzing the variable region of the cp gene of *V. mongolicum* and other *Vincetoxicum*, we found that the variable sequences of *rpoC1-rpoB*, *ycf4-cemA*, *ndhF*, *ndhF-rpl32*, and *rpl32-ccsA* fragments were highly significant, which could be targeted as the DNA barcodes for evidence of *V. mongolicum* and its relatives in Apocynaceae. Maximum-likelihood (ML) phylogenetic tree analysis elucidated that *V. mongolicum* was sister to *V. pycnostelma* with strong support. Our results provide useful information for future phylogenetic studies and plastid super-barcodes of the family Apocynaceae.

*Vincetoxicum mongolicum* Maxim. 1876, is a poisonous herb on the grassland, and also a perennial medicinal plant, widely distributed in the Loess Plateau region of China (Jiang and Li, 1987). The scientific name of *V. mongolicum* was merged during the correction of the Angiosperm Phylogeny Group (APG) classification system, previously named “Lao Gua Tou” or “Niu Xin Pu Zi” under the scientific name of *Cynanchum komarovii* Al. Iljinski, is one of the indicator plants of desertification in arid or semi-arid regions (Chase et al., 2016; Wang et al., 2017). Therefore, the documented studies associated with *C. komarovii* are in fact studies of *V. mongolicum*. It has been reported that *C. komarovii* is a traditional analgesic drug, and has a role in promoting blood circulation, relieving pain, and reducing inflammation (Lu et al., 1997). With the continuous development of medical detection technology, new chemical components of *C. komarovii* have been gradually discovered, such as two new C_21_ steroidal glycosides (Zhao et al., 2018), volatile oil (Wang and Yang, 2010), alkaloids (Wang, 2019), and antibacterial ingredients (Bi et al., 2014), etc. In ethnic minority areas of China, *C. komarovii* is often used to treat various painful diseases and is regarded as an important ethnodrug (Jia *et al.*, 2015). Recent studies have found that the total alkaloids of *V. mongolicum* have obvious analgesic activity (Wang *et al.*, 2022), which provides a scientific explanation for the effective folk medication events in the past ethnic areas of China. Although studies of the related species of *V. mongolicum* have been developed in recent years, such as endophyte (Bi *et al.*, 2014; Dickinson et al., 2021), taxonomy (Xiong et al., 2019; Jackson and Amatangelo, 2021; Yu et al., 2021; Ye et al., 2022), there are still many fields worth exploring. Hence, there is still a lack of evidence on the development and phylogenetic status of the medicinal resources of *V. mongolicum*. The sequence of divergent events of gene segments in plastids provides convenience for us to understand the phylogeny and classification of plants and is also the key to marking the phylogenetic status of species (Mishra et al., 2016; Van Do et al., 2021). Given the perspective of phylogeny, we determined the complete chloroplast (cp) genome of *V. mongolicum* and analyzed the genetic variation and clustering to provide a reference for subsequent studies.

Fresh leaves of *V. mongolicum*were collected from the Mu Us Sandy desert in eastern Ningxia, China (38°4′56.66 N, 106°32′56.47 E, alt. 1147 m), and samples are dried with silica gel. The voucher specimen (640181VM-2022003LY) was deposited at the herbarium of the Institute of Forestry and Grassland Ecology, Ningxia Academy of Agriculture and Forestry Science (http://www.nxaas.com.cn/, Wangsuo Liu, email: liuwangsuo@sina.com). Genomic DNAs were extracted by the modified CTAB method of Stefanova et al. (2013). The complete cp genome was sequenced by Illumina Hiseq 2500 from Biomarker Technologies Corporation. About 143,846,691 clean reads were obtained and used to assemble into reference sequences after trimming using the GetOrganelle online tool (http://github.com/Kinggerm/GetOrganelle), with SPAdes3.11.0 as assembler (Nurk et al., 2013). The genes were annotated using the GeSeq webserver (https://chlorobox.mpimp-golm.mpg.de/geseq.html) (Tillich et al., 2017). The cp genome of *V. mongolicum* was mapped using the online tool OGDRAW (https://chlorobox.mpimp-golm.mpg.de/OGDraw.html) (Greiner et al., 2019). Simple sequence repeat (SSR) analysis of *V. mongolicum* was performed by the web server MIcroSAtellite (https://webblast.ipk-gatersleben.de/misa) (Beier et al., 2017), and the SSRs parameters 10, 6, 5, 5, 5, and 5 represent the thresholds of mononucleotide, dinucleotide, trinucleotide, tetranucleotide, pentanucleotide, and hexanucleotide, respectively.

The sequence of the *V. mongolicum* complete cp genome has been submitted to the NCBI database with the accession number ON854661. Setting *V. rossicum* (KF539854) as the reference, we downloaded the cp genome of *V. pycnostelma* (OK271107), *V. forrestii* (NC060305), *V. versicolor* (NC052877), *V. junzifengense* (NC062962), *V. shaanxiense* (NC0046785), and *V. hainanense* (NC051946) from the NCBI database. The cp genome of *V. mongolicum* (ON854661) was compared with the above-mentioned six species, using the online tool mVISTA with a shuffle-LAGAN model (http://genome.lbl.gov/vista/mvista/submit.shtml) (Frazer et al., 2004). To better locate the phylogenetic position of *V. mongolicum*, the complete cp genome of 33 related species in Apocynaceae and 2 outgroups were downloaded from the NCBI database and aligned with *V. mongolicum* via MAFFT 7.037 (Katoh and Standley, 2013). A maximum-likelihood tree analysis was performed using the Tamura-Nei model of MEGA X with 1000 bootstrap replicates (Kumar et al., 2018) based on 36 species.

The complete cp genome of *V. mongolicum* has is a typical circular shape with a length of 160,157 bp, including a large single copy (LSC) region of 91,263 bp, a pair of inverted repeats (IRa and IRb) region of 23,892 bp, and a small single copy (SSC) region of 21,110 bp. The GC content of the *V. mongolicum* cp genome accounted for 37.8%, and the GC content in IR (43.6%) regions was higher than that of LSC (36.0%) and SSC (32.1%) regions. The cp genome of *V. mongolicum* displayed 131 genes, including 86 protein-coding genes, 37 tRNA genes, and 8 rRNA genes. In the IR region, there were 18 duplicated genes identified including 7 protein-coding genes (*ndhB*, *rpl2*, *rpl23*, *rps12*, *rps7*, *ycf15*, *ycf2*), 7 tRNA genes (*trnA-UGC*, *trnI-GAU*, *trnI-CAU*, *trnL-CAA*, *trnN-GUU*, *trnR-ACG*, *trnV-GAC*), and 4 rRNA genes (*rrn16S*, *rrn23S*, *rrn4.5S*, *rrn5S*) (Figure 1). The length of sequence repeats in each region of the cp genome plays an important role in the diversity and inheritance of cp genome recombination (Yang et al., 2019). SSR polymorphism has been widely used in species identification and genetic diversity research (Powell et al., 1995). In this study, a total number of 66 SSRs were identified, including 60 mononucleotides, 5 dinucleotides and 1 trinucleotide. The majority of SSRs were mononucleotides, accounting for 91%, and the main bases were A and T. The result was consistent with the findings of the other plants (Li et al., 2020a; Wei et al., 2020; Alzahrani et al., 2021; Luo et al., 2021). SSRs were mainly located in the LSC region, accounting for 56%, followed by the SSC region, accounting for 38%, while in the IR region, accounting for 6% (Figure 2A). Most repeats detected were mononucleotide. In the LSC region, 37 mononucleotide, four dinucleotide, and one trinucleotide repeat detected; in the SSC region, 19 mononucleotide and one dinucleotide repeat were found while only four mononucleotide repeats were identified in the IR region (Figure 2B-D), this was likely due to genetic polymorphism of *V. mongolicum*.

**Figure 1 -:**
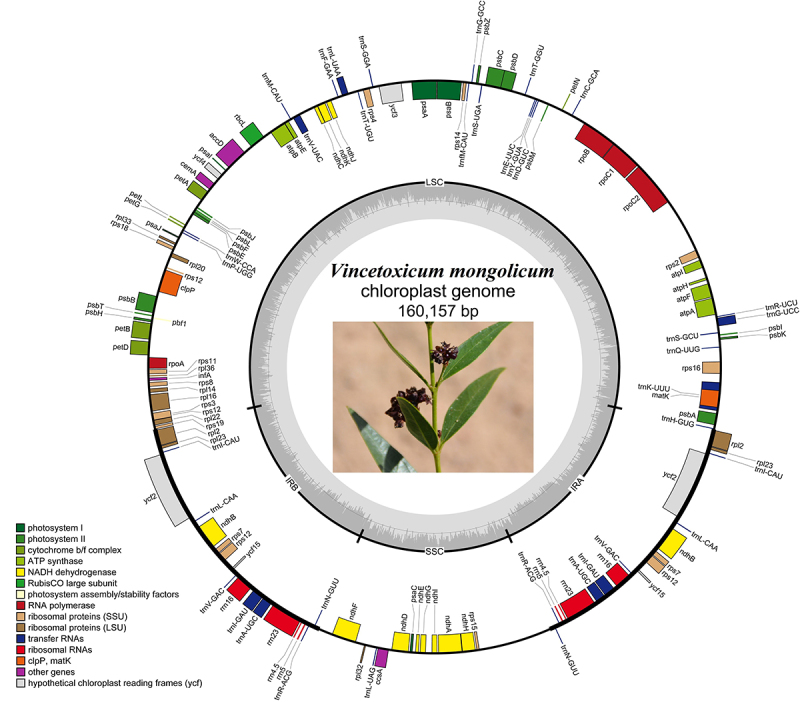
Map of the *V. mongolicum* chloroplast genome. Genes shown inside the circle indicate that the direction of transcription is clockwise, while those outside the circles are counterclockwise. Different groups of functional genes are represented in different colors. The GC content is displayed in the dashed area in the inner circle.

**Figure 2 -:**
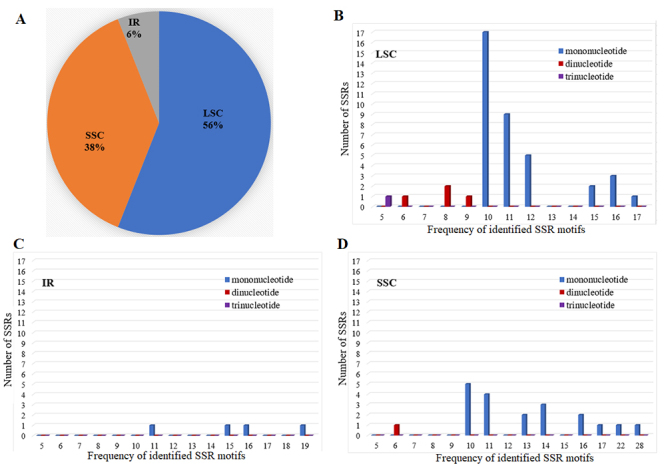
Simple sequence repeats (SSRs) in the cp genome of *V. mongolicum*. A: The proportion of SSRs in the LSC, SSC, and IR regions. B: Presence of nucleotide in the LSC regions. C: Presence of nucleotide in the IR regions. D: Presence of nucleotide in the SSC regions.

Highly variable regions of the cp genome are often used as DNA barcodes for plant classification. DNA barcoding can provide an important evidence for plant phylogeny and classification, genetic variants commonly used as DNA barcodes, including *petA-psbJ*, *rps16-trnQ*, *ndhC-trnV*, *ycf1*, *ndhF*, *trnK*, *rpl32-trnL*, *trnH-psbA*, *rpoB-trnC*, *psbE-petL*, and *rbcL-accD* (Dong et al., 2012). The sequence divergent graphs of the seven *Vincetoxicum* cp genomes were drawn by using the online tool mVISTA (Figure 3). The results showed that the *rps16-psbK* gene segment was significantly different in all the complete cp genomes of the seven species of *Vincetoxicum*, which could be used as a key genetic indicator for their phylogenetic classification. The *rps16-psbK* gene fragment was found to be a hypervariable region in the cp genomes of almond (Wang et al., 2020) and *Crataegus* (Wu et al., 2022) species and could be used as a divergent region in the genus of *Vincetoxicum* in this study. 

**Figure 3 -:**
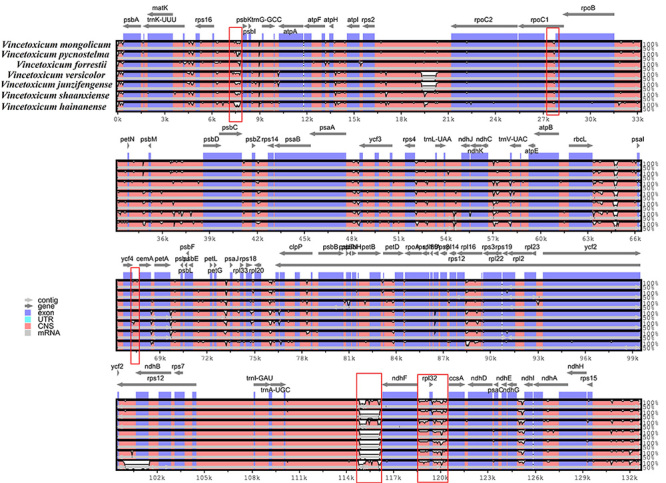
Comparison of the cp genome sequences of *V. mongolicum*, *V. pycnostelma*, *V. forrestii*, *V. versicolor*, *V. junzifengense*, *V. shaanxiense*, and *V. hainanense*, generated with mVISTA. Grey arrows above the alignment represent the direction and position of each gene. Areas of significant variation are marked with red boxes. Genic and intergenic regions were indicated by blue and red areas, respectively. The vertical scale represents the percentage of identity and ranges from 50 to 100%.

In general, the non-coding regions displayed great divergence, and the coding regions were relatively conservative. In the sequences of the cp genome of *V. mongolicum*, we found five different divergent regions of *rpoC1-rpoB*, *ycf4-cemA*, *ndhF*, *ndhF-rpl32,* and *rpl32-ccsA*, which can be recommended for DNA barcoding of *V. mongolicum* for the evolutionary classification. These results suggest that the sequence regions of *rpoC1-rpoB*, *ycf4-cemA*, *ndhF*, *ndhF-rpl32,* and *rpl32-ccsA* could be targeted as the DNA barcodes of *V. mongolicum* in phylogenetic evolution. *Ycf4-cemA* is a highly variable locus in the cp genome of most plants, and a study showed that the sequencing segment of *ycf4-cemA* could be used as a key cp gene marker for the evolutionary classification of *Acer* in Aceraceae (Ma et al., 2019). We found that this divergent region of *V. mongolicum* existed in many plants such as *Morella rubra* (Liu et al., 2017), *Angelica polymorpha* (Park et al., 2019), *Coffea arabica* (Samson et al., 2007), *Impatiens* (Luo et al., 2021), *Mangifera* (Niu et al., 2021), *Aconitum* (Park *et al.*, 2017), *Arnebia* and *Lithospermum* (Park *et al.*, 2020), *Justicia* (Zhou et al., 2021), and *Populus* (Zong et al., 2019), indicating the variation of *ycf4-cemA* gene region is a common event in plants. The divergent sequence of *ndhF* gene fragment appeared in medicinal plants of *Dolomiaea* (Shen et al., 2020), *Rheum* (Xin et al., 2022), and *Crataegus* (Wu et al., 2022), supporting our findings of a highly differentiated *ndhF* segment of the photosynthetic system gene in the cp genomes of *Vincetoxicum*. In this study, two highly variable regions of *ndhF-rpl32* and *rpl32-ccsA* were found in the cp genome of *V. mongolicum*, which were also marked and identified as the DNA barcodes in plants such as *Pterocarpus* (Jiao et al., 2019), *Stipa* (Krawczyk et al., 2018), *Ardisia* (Xie et al., 2021), and *Alpinia* (Li *et al.*, 2020a). Moreover, the variable loci of *ndhF-rpl32* and *rpl32-ccsA* mostly occurred in the location of the SSC region of the *Dioscorea*, *Digitaria,* and *Pennisetum* species (Scarcelli et al., 2011). The high gene divergence will be meaningful in future studies involving population genetics and origin of phylogeny for *V. mongolicum*.

The phylogenetic analysis was conducted based on the 36 complete cp genomes by maximum likelihood (ML), it suggested that *V. mongolicum* was sister to *V. pycnostelma* with strong support (Figure 4). The 34 species from Apocynaceae showed a long genetic distance from the two outgroups of Gentianaceae. The evolutionary status of *Vincetoxicum* (Xiong et al., 2019; Ye et al., 2022), *Biondia* (Rao et al., 2018; Guan and Zhang, 2019), and *Wrightia* (Li et al., 2020b) in Apocynaceae family has been reported. Our study firstly reported the complete cp genome characteristics and the high variation regions of *V. mongolicum*, and provides an analysis of the phylogenetic relationship of the genus *Vincetoxicum*, which would provide meaningful information for future evolutionary studies and plastid super-barcodes of the family Apocynaceae.

**Figure 4:**
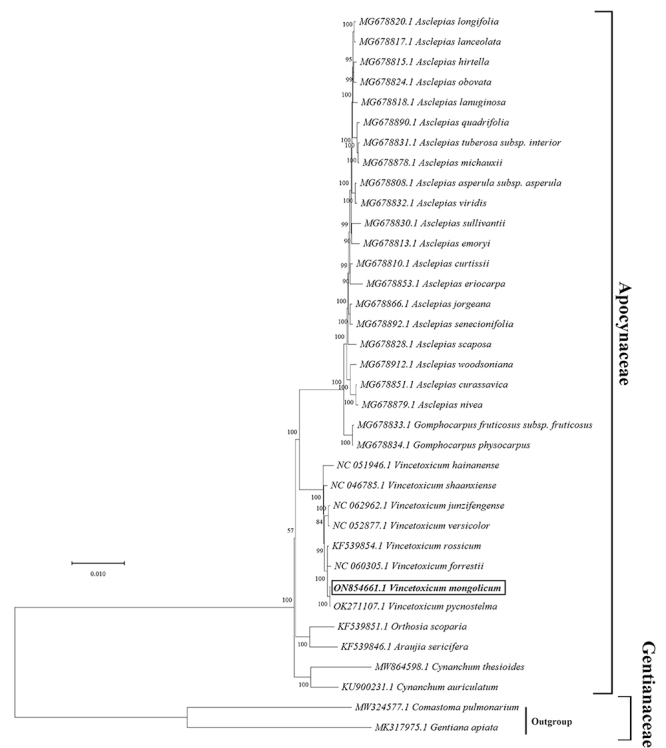
Maximum-likelihood tree constructed using MEGA X based on 36 complete cp genomes. The number on each node represents bootstrap values.
